# Pipeline for Analyzing Lesions After Stroke (PALS)

**DOI:** 10.3389/fninf.2018.00063

**Published:** 2018-09-24

**Authors:** Kaori L. Ito, Amit Kumar, Artemis Zavaliangos-Petropulu, Steven C. Cramer, Sook-Lei Liew

**Affiliations:** ^1^Neural Plasticity and Neurorehabilitation Laboratory, University of Southern California, Los Angeles, CA, United States; ^2^Imaging Genetics Center, Mark and Mary Stevens Neuroimaging and Informatics Institute, Keck School of Medicine, University of Southern California, Marina del Rey, CA, United States; ^3^Department of Neurology, University of California, Irvine, Irvine, CA, United States

**Keywords:** stroke, big data, lesion analysis, lesion load, MRI imaging, neuroimaging, stroke recovery

## Abstract

Lesion analyses are critical for drawing insights about stroke injury and recovery, and their importance is underscored by growing efforts to collect and combine stroke neuroimaging data across research sites. However, while there are numerous processing pipelines for neuroimaging data in general, few can be smoothly applied to stroke data due to complications analyzing the lesioned region. As researchers often use their own tools or manual methods for stroke MRI analysis, this could lead to greater errors and difficulty replicating findings over time and across sites. Rigorous analysis protocols and quality control pipelines are thus urgently needed for stroke neuroimaging. To this end, we created the Pipeline for Analyzing Lesions after Stroke (PALS; DOI: https://doi.org/10.5281/zenodo.1266980), a scalable and user-friendly toolbox to facilitate and ensure quality in stroke research specifically using T1-weighted MRIs. The PALS toolbox offers four modules integrated into a single pipeline, including (1) reorientation to radiological convention, (2) lesion correction for healthy white matter voxels, (3) lesion load calculation, and (4) visual quality control. In the present paper, we discuss each module and provide validation and example cases of our toolbox using multi-site data. Importantly, we also show that lesion correction with PALS significantly improves similarity between manual lesion segmentations by different tracers (*z* = 3.43, *p* = 0.0018). PALS can be found online at https://github.com/npnl/PALS. Future work will expand the PALS capabilities to include multimodal stroke imaging. We hope PALS will be a useful tool for the stroke neuroimaging community and foster new clinical insights.

## Introduction

Characterizing the relationship between brain structure and function is an important step in identifying and targeting biomarkers of recovery after stroke (Dimyan and Cohen, 2011). As stroke is heterogeneous in both its anatomical and clinical presentation, it is often difficult to draw generalizable inferences with typical sample sizes. Moreover, many stroke research groups have traditionally operated in silos (Hachinski et al., 2010). This poses a problem for scientific reproducibility, as different research groups have various in-house analytic processes and pipelines that are often not transparent (Gorgolewski and Poldrack, 2016). In recent years, big data approaches have emerged and been embraced in the neuroimaging field (Milham, 2012). This offers new hope for discovery of otherwise difficult-to-detect neural patterns that hold promise for promoting advanced therapeutic techniques (Feldmann and Liebeskind, 2014; Huang et al., 2016). While promising in their potential to overcome the problem of heterogeneity in stroke research, big data approaches to research come with their own challenges, especially with respect to combining data across sites, and managing and analyzing such large quantities of data (Van Horn and Toga, 2014). Particularly for the analysis of data from persons with stroke, there is a pressing need for the development of reproducible image processing and analysis pipelines that properly incorporate the lesion to promote collaborative efforts in the analysis of large stroke datasets.

The semiautomatic brain region extraction (SABRE) pipeline is one such example of an image processing pipeline for lesion analysis that has been made open-source (Dade et al., 2004). The SABRE pipeline integrates currently existing software, such as FSL and ANTs to allow for volumetric profile of regionalized tissue and lesion classes, while emphasizing quality control (Avants et al., 2009; Jenkinson et al., 2012). However, the SABRE pipeline was not specifically developed for stroke MRIs, and requires multi-modal inputs, which are not commonly available for research on chronic stroke.

**FIGURE 1:**
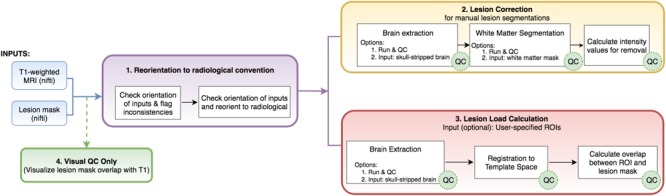
Analysis pipeline. PALS takes in a minimum of two inputs (in blue): a T1-weighted MRI and a lesion mask file and has four main modules: (1) reorientation to radiological convention, (2) lesion correction, (3) lesion load calculation, and (4) visual QC. Users can choose to perform any or all of the main modules. White boxes indicate processing steps used in the pipeline. Green “QC” circles indicate that PALS will create a quality control page for that processing step. ROIs, regions of interest.

To this end, we created the Pipeline for Analyzing Lesions after Stroke (PALS; DOI: https://doi.org/10.5281/zenodo.1266980), an open-source analysis pipeline with a graphical user interface (GUI) to facilitate reproducible analyses across stroke research sites using a single modality—a T1-weighted MRI, which is the most commonly available for chronic stroke research. Our goal is to improve the standardization and analysis of stroke lesions and to encourage collaboration across stroke research groups by creating a flexible, scalable, user-friendly toolbox for researchers. PALS has four modules integrated into a single analysis pipeline (**Figure 1**, bolded text): (1) reorientation of image files to the standard radiological convention, (2) lesion correction for healthy white matter, which removes voxels in the lesion mask that are within a normal intensity range of white matter, (3) lesion load calculation, which calculates the number of voxels that are overlapping between the lesion and a specified region of interest, and (4), visual quality control (QC), which creates HTML pages with screenshots of lesion segmentations and intermediary outputs to promote visual inspection of data at each analysis step. Notably, researchers should use a method of their choice to generate the initial lesion masks for their dataset before using PALS. We provide a comprehensive review of all existing automated lesion segmentation methods (Ito, Kim, and Liew, under review), and note that the gold standard is still manual lesion segmentation. However, once lesion masks are generated, whether through automated or manual methods, the PALS pipeline will facilitate quality control and additional analyses using the lesion masks.

The rationale for each step was informed by both existing literature as well as current attempts to combine stroke data collected across multiple sites (Liew et al., 2018). In this report, we will first review the rationale for each of these features, then discuss the implementation of the features, and finally present results from using the toolbox on multi-site data. The compiled toolbox, source code, and instructions can be freely accessed at our Github repository^1^.

## Main Features: Rationale

**FIGURE 2:**
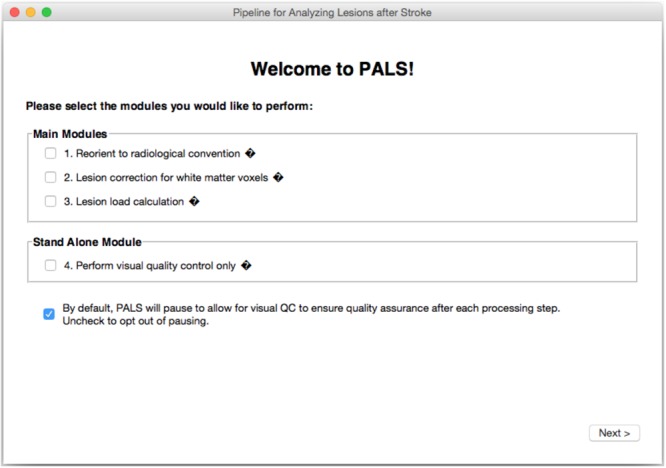
PALS Interface. PALS has a simple and intuitive graphical user interface (GUI) which allows back and forth navigation and easy selection of modules. Additionally, the interface has tool tip icons (indicated by small black diamonds with question marks), which provide a brief helper text about each function when the user hovers over the tool tip icon.

PALS features a GUI-based navigation system for ease of use (**Figure 2**). Any combinations of the four modules (reorientation to radiological convention, lesion correction, lesion load calculation, and visual quality control) can be selected and the entire pipeline will run automatically.

### Reorientation to Radiological Convention

Inconsistent orientation of images within a dataset is a common and serious issue in image processing. Neurological and radiological orientations are both widely used conventions for storing image information (Brett et al., 2017). Whereas the neurological convention stores a patient’s left side on the left part of the image, the radiological convention stores left side information on the right side of the image. The convention in which image information is stored can vary between scanners or even acquisition parameters, such that some images are stored in the radiological convention, and others are stored in the neurological convention. Moreover, commonly used neuroimaging processing tools display and store information in different ways, which can lead to orientation inconsistencies. For example, FSL and FSLeyes by default displays images in radiological convention (Jenkinson et al., 2012), the SPM display utility by default displays images in neurological convention (Penny et al., 2011), and MRIcron allows users to switch between the orientations (Rorden and Brett, 2000). If image labels are inconsistent or incorrect, analyses may be negatively impacted since one may be incorrectly flipping the two sides of the brain (Duff, 2015). This is particularly problematic for stroke neuroimaging research, as one may mislabel the hemisphere of the stroke lesion. As such, image orientation needs to be carefully considered especially for large collaborative efforts, when data has been collected from multiple sites. We thus built a simple, optional module to convert all image inputs to the radiological convention prior to performing any subsequent step to harmonize data across sites. We recommend use of this module with all datasets.

### Lesion Correction for Healthy White Matter Voxels

While many automated approaches have been developed for lesion segmentation, manual segmentation remains the gold standard for tissue labeling and continues to be the benchmark for automated approaches (Fiez et al., 2000; Maier et al., 2017). Yet, depending on the size and location of the lesion, manual lesion segmentation could be a highly time- and labor-intensive process. This becomes particularly challenging for large, multi-site collaborative efforts, as having larger datasets places an increasing demand on skilled manual labor. As such, multiple individuals are often trained to perform lesion segmentations to distribute the heavy labor demands. However, the wide variability in lesion characteristics as well as inter-subjective differences in the way that lesions are defined may introduce potential inconsistencies in the manual lesion segmentation process (Fiez et al., 2000). Lesion correction for healthy white matter voxels is one method proposed to decrease subjective differences in the manual definition of lesions (Riley et al., 2011). The lesion correction aims to correct for intact white matter voxels that may have been inadvertently included in a manually segmented lesion mask. This is done by removing voxels in the lesion mask that were within the intensity range of a healthy white matter mask. We previously created a semi-automated toolbox to address this (SRQL toolbox; Ito et al., 2017). However, it required manual delineation of a white matter mask for each subject. Here, we integrated an updated version of the SRQL toolbox as an optional lesion correction module that improves on the SRQL toolbox by taking advantage of automated white matter segmentation in FSL. We note that we recommend use of the lesion correction module only on manually segmented lesions, and not on automated segmentations, as evidenced in our validation work below. Furthermore, careful visual inspection of white matter segmentation masks should be completed prior to using this module.

### Lesion Load Calculation

Currently, one of the main goals of stroke research is to identify biomarkers for recovery, which can help identify patient subgroups and predict which treatments would be most beneficial for different patient subgroups (González, 2006; Cramer, 2010; Stinear, 2017). Studying the anatomy and precise location of stroke lesions is one potential avenue for drawing clinically meaningful inferences about recovery. Specifically, the structural integrity of white matter motor pathways, which has been measured as the overlap of the lesion with a corticospinal (CST) tract template, has been associated with motor performance (Zhu et al., 2010; Riley et al., 2011; Stinear, 2017), and it has been suggested that good recovery of motor function is largely reflective of spontaneous processes that involve the ipsilesional motor pathway (Byblow et al., 2015). In fact, it has been shown that both initial motor impairment and long-term motor outcome are dependent on the extent of CST damage, and the extent of white matter damage had greater predictive value than lesion volume (Puig et al., 2011; Feng et al., 2015). The extent of CST damage has been developed into an imaging biomarker as the weighted CST lesion load, which is calculated by overlaying lesion maps from anatomical MRIs with a canonical, atlas-based CST tract (Riley et al., 2011). Here, we built a module to calculate the CST lesion load using T1w MRIs, and validate use of our module against a similar lesion load calculator (Riley et al., 2011). However, as it is likely that other motor and non-motor regions in the brain may also be predictive of motor or cognitive recovery (Crafton et al., 2003; Rondina et al., 2017), we have extended the lesion load module to analyze lesion overlap with corticospinal tract or other cortical and subcortical structures and tracts, based on regions of interest from the FreeSurfer software and sensorimotor area tract template (S-MATT; Archer et al., 2017) packages, respectively.

### Visual Quality Control

To analyze large quantities of data efficiently, most neuroimaging processing steps are now automated. Yet the presence of a stroke lesion substantially increases the susceptibility to image preprocessing errors (Andersen et al., 2010; Siegel et al., 2017). The accuracy of each image processing step, including but not limited to lesion segmentation, brain extraction, and normalization, could impact subsequent downstream processing and analyses. Therefore, visual inspection of automated output is imperative for lesion analyses. To this end, we encourage visual inspection of data for quality data assurance by integrating the creation of quality control review pages for each preprocessing step that PALS requires. PALS is designed to pause after each intermediary step and ask the user to inspect the data and provide manual input on whether each subject’s output passes visual inspection (which can be marked in a checkbox under each individual). From there, PALS will only perform subsequent analyses on subjects that pass the visual inspection. If, however, users wish to run all subjects through the entire pipeline without pausing, they are given the option to do so, but are highly encouraged to visually inspect all analyses steps after completion.

For users who simply wish to efficiently visualize lesion masks and do not wish to run other modules, PALS also offers the visual quality control feature as a stand-alone tool.

## Basic Structure of Pals Directories

**FIGURE 3:**
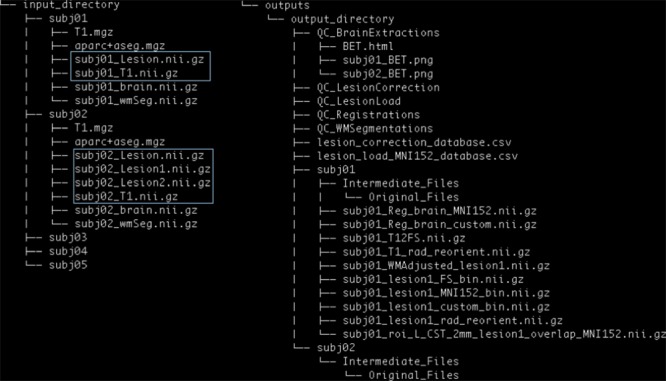
PALS data structure. The user is expected to provide an input directory with subdirectories as shown above. Files in blue boxes are necessary inputs. The user is also expected to provide the path to an output directory, and PALS will create all other directories and files under the output directory.

PALS requires the user to specify the path to an *Input Directory* and an empty *Output Directory* (**Figure 3**).

### Inputs

The *Input Directory* must contain separate *Subject Directories* for each subject. Each *Subject Directory* must at minimum contain: the subject’s T1-weighted anatomical image in NifTI format, and one or more corresponding lesion masks, also in NifTI format. Importantly, all inputs should be in valid NifTI format and have the same image dimensions within each subject. T1 anatomical images for all subjects must contain the same T1 image identifier (e.g., T1 images for the first and second subject should be subj1_T1.nii.gz and subj2_T1.nii.gz, respectively); similarly lesion masks for all subjects must contain the same lesion mask identifier (e.g., subj1_Lesion.nii.gz and subj2_Lesion.nii.gz). If any subject has multiple lesions, each additional lesion mask must contain the lesion identifier, appended by the index, beginning with one for each additional lesion (e.g., subj1_Lesion1.nii.gz; see blue boxes in **Figure 3**).

Additionally, if the user chooses to run the Lesion Correction and/or Lesion Load Calculation modules, they are given the option to include the following files in each *Subject Directory*: a brain mask file (NifTI) and a white matter segmentation file (NifTI). If these steps have already been performed, brain extraction and white matter segmentation can be skipped during subsequent analyses. One caveat of this is that the same option must be implemented for all subjects in a given analysis pipeline. That is, the user cannot choose to skip brain extraction for only one subject; they would have to skip the step and provide their own brain mask files for all subjects.

If the user has already performed FreeSurfer cortical and subcortical segmentation for each subject, they may use subject-specific ROIs derived from FreeSurfer for lesion load calculation. If so, the user will be required to provide a (1) T1.mgz and (2) aparc + aseg.mgz parcellation and segmentation volume file from FreeSurfer outputs in each *Subject Directory*. The same caveat of pursuing the same option for all subjects applies.

### Outputs

To encourage reproducible analysis, PALS also automatically creates time-stamped log files indicating selected options, inputs, and all processing steps each time it is run. These log files can be found in the source directory for PALS under the logs directory. This directory will only be created after the first run of PALS.

The general structure of the *Output Directory* will look similar to that of the *Input Directory*, with a separate directory created for each subject. Each new *Subject Directory* will contain the final outputs of the selected modules (e.g., white matter intensity adjusted lesion masks for the lesion correction module), and a subdirectory called *Intermediate_Files*, in which outputs from intermediary processing steps will be stored. Within the *Intermediate_Files* directory will be an *Original_Files* directory, which will contain a copy of all input files for the subject. Please see our github page^2^ for a detailed description of each output file.

The *Output Directory* will additionally contain separate *QC* directories for each intermediary step taken (e.g., *QC_BrainExtractions* for the brain extraction step). These *QC* files will contain screenshots for each subject, and a single HTML page for manual visual control.

Finally, if the lesion correction and/or lesion load modules are selected, the *Output Directory* will also contain CSV files with information on the lesion (e.g., number of voxels removed during lesion correction, and percentage of lesion-ROI overlap per subject).

## Implementation

### Dependencies

PALS was built in Mac OSX on Python 2.7 and requires pre-installation of FSL. Separate installation of FSLeyes is necessary only if a version of FSL older than 5.0.10 is installed. FreeSurfer installation is necessary only if the user desires to use subject-specific FreeSurfer segmentations for the lesion load calculation module (see more information on lesion load calculation below).

PALS is compatible with Unix and Mac OS operating systems. For first-time users, PALS will ask users for the directory path to FSL binaries. While we note that only 9 MB of space is needed for PALS installation (not including its dependencies), the total amount of space used for outputs created by the program will vary widely depending on the operations and number of subjects selected. Minimally, we recommend that 54 MB is allocated per subject, assuming only one ROI is selected for lesion load calculation, to run all operations.

### Modules

#### Reorienting to Radiological Convention

The purpose of the reorient to radiological module is to make sure that lesion masks are in the same convention as the anatomical brain file, since some software used to create lesion masks may flip the orientation of the lesion file. Additionally, this module attempts to homogenize the orientation of files across subjects, especially when combining data across sites. Importantly, this module assumes that the conversion from DICOM to NifTI format was performed correctly. There should be no errors in data storage and no missing information in the NifTI header.

The reorientation module first checks the orientation of the T1 anatomical and lesion mask images. If they are already in the radiological convention as indicated by the image header, the image convention is conserved. If both T1 and lesion mask images are found to be in the neurological convention, the image data and image header for both the T1 anatomical image and associated lesion masks are changed to the radiological convention, using FSL commands *fslswapdim* and *fslorient*, respectively^3^. If, however, the T1 and lesion masks are not in the same convention, PALS flags the subject and does not perform subsequent analyses on that subject. We recommend that the user perform a thorough check of all flagged subjects to verify that image orientations are correct by running FSL command *fslorient* on flagged images.

If the user has also provided additional optional inputs, such as a skull-stripped brain and/or white matter mask, those images are also reoriented to the radiological convention if they are not already. Finally, the FSL command *fslreorient2std* is applied on all images to reorient images to match the orientation of a standard T1-weighted template image (MNI152).

#### Lesion Correction for Healthy White Matter Voxels

The basic steps that SRQL, the original toolbox we created for lesion correction, implements for white matter lesion correction are outlined elsewhere (Ito et al., 2017). However, as several steps have been modified and updated for PALS, we describe the steps in detail here.

First, the intensity of each subject’s T1 structural image is scaled to a range within 0 to 255 (intensity normalization). Skull stripping using FSL’s Brain Extraction Toolbox (BET) and automated white matter segmentation using FSL’s Automated Segmentation Tool (FAST) are then performed (Smith, 2002). The user is given an option to skip the skull-stripping and segmentation steps if he or she specifies that these steps have already been performed. If skull-stripping and/or white matter segmentation are performed, PALS will create a quality control page and the program will pause for the user to perform a visual inspection of each brain extraction/white matter segmentation.

Next, intensity normalized values from the T1 image (step 1) are projected onto the white matter segmentation as well as the binarized lesion mask, and the mean white matter intensity value is calculated from the white matter segmentation.

To calculate the upper and lower bounds for white matter intensity removal, the percent intensity for removal is first specified by the user. A default value of 5% is built into the toolbox. The specified percentage for removal is then converted to a 0 to 255 scale and divided by 2. This value is added to and subtracted from the mean white matter intensity value, such that:

$$\begin{array}{c} Intensity values to be removed=\mathrm{mean}\pm \frac{\left(255*specified percentage\;\%\right)}{2} \end{array}$$

Following this calculation, any voxels with intensity values within this range in the T1-projected lesion mask are removed, thereby removing voxels in the lesion mask that are within the specified intensity range of healthy white matter for that individual. As the last step, the white matter adjusted lesion mask file is binarized as a final lesion mask.

After lesion correction has been completed for all subjects, a CSV file containing information about the number of voxels removed for each subject’s corrected lesion is created along with a quality control page for visual inspection of the effect of lesion correction on the lesion. The impact of using the lesion correction module is reported in validation (section IV), where we show that lesion correction decreases inter-individual variability on manual segmentations, but does not improve upon automated segmentations.

#### Lesion Load Calculation

The lesion load calculation module computes the amount of lesion-ROI overlap with minimal input from the user. Notably, the user does not need to register or reslice regions of interest (ROI) to native space prior to using the lesion load module in PALS—PALS automatically normalizes all native space lesion masks and anatomical files to match that of the ROI. We offer several options for selecting ROIs in calculating lesion load based on commonly-used conventions for ROI analysis (Poldrack, 2007). (1) PALS comes with a set of default anatomical ROIs, all of which have been converted to standard 2 mm MNI152 space, including bilateral corticospinal tract ROIs (Riley et al., 2011), FreeSurfer subcortical and cortical ROIs (Fischl, 2012), and sensorimotor area tract ROIs (S-MATT; Archer et al., 2017). (2) We also allow calculation of lesion load using subject-specific FreeSurfer cortical and subcortical segmentations, if the user indicates that they have already performed FreeSurfer and have FreeSurfer-derived aparc + aseg.mgz and T1.mgz files for each subject. (3) Lastly, we give users the option of providing their own regions of interest to calculate lesion load. This option requires that the user also provides the standard space template of the regions of interest so that PALS can convert subject files to the ROI space.

After the ROIs are specified by the user, all ROIs are binarized, and lesion masks and T1 images are registered to the ROI space, whether it is MNI152 (default ROIs), FreeSurfer space, or user-defined. At this point, the program will pause again and have the user perform a visual inspection of the registrations to confirm that the normalization looks appropriate. Lesion masks are also binarized (such that only voxels within the lesions have a value of 1 and all other voxels have a value of 0), and summed with the voxel values of each binarized ROI mask using the FSL command *fslmaths*, so that regions that are overlapping between the lesion and ROI have a value of 2. Next, to obtain the mask of the lesion-ROI overlap, a threshold is applied to the combined lesion-ROI mask, such that anything below a value of 2 is zeroed. Finally, the lesion-ROI overlap mask is used to calculate the total percentage of overlap between the lesion and ROI, calculated as:

$$\begin{array}{c} Percentage overlap=\frac{(overlap volume between lesion and ROI)}{ROI volume} \end{array}$$

The percentage overlap between the lesion and ROI is then saved into a CSV file containing lesion load information for all subjects, and a quality control page is created for visual inspection of lesion load performance.

#### Quality Control Webpages

PALS uses the FSL module *fsleyes*^4^ to render screenshots of each subject’s brain extraction overlaid on its corresponding T1-weighted image. The screenshots will display the overlays along the three orthogonal planes. These screenshots are concatenated into a single HTML page for review. Below each screenshot is a checkbox for the user to indicate whether the subject’s brain extraction passes visual inspection.

**FIGURE 4:**
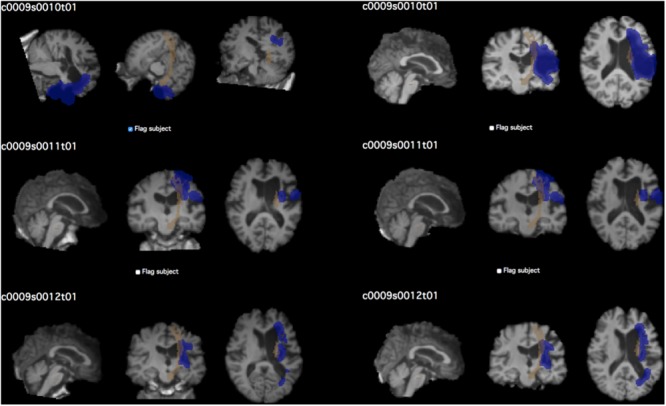
Example of quality control page. The QC page shown above was used to assess the quality of CST lesion load calculations in the test dataset. Each subject’s screenshot is displayed with a checkbox below for flagging subjects that do not pass visual inspection. Left, prior to performing QC; right, after correcting brain extraction. The first subject in particular demonstrates the vast difference that quality inspection makes. Brown shows the CST ROI; blue shows the lesion mask of each subject.

The same process was used to create quality control pages for each subject’s white matter segmentation mask and registered brain masks. For lesion masks, both the final white matter adjusted mask as well as the original manually traced lesion mask were overlaid onto the T1-image for comparison of lesions before and after lesion correction. For lesion load calculations, the lesion mask is overlaid on the selected region of interest for calculating the lesion load (see **Figure 4**).

## Validation

### Reorient to Radiological

Here, we validated that this tool performs each function correctly. For this evaluation, we simply wanted to test as many cases as possible, and used a combined dataset of 355 MRIs and lesion masks from 12 research sites [11 from the Anatomical Tracings of Lesions after Stroke (ATLAS) database, and one additional from a collaborator; Liew et al., 2018]. We checked that PALS correctly flagged all 30 subjects whose lesion mask and anatomical T1 files had mismatching orientations. For all other images that were not flagged, the module corrected identified images in the neurological orientation and transformed them to radiological orientation (see **Supplementary Table S1**).

We next simulated cases to confirm that PALS also correctly identified subjects with mismatched orientations in optional inputs (e.g., where a brain mask and/or white matter mask file are provided by the user in addition to the necessary T1 and lesion mask files). Additionally, we created a case in which all but one subject contained the additional optional inputs. We checked that PALS was able to correctly identify when orientations of inputs were mismatched for any subjects, and overrides the user input to skip brain extraction and/or white matter segmentation if a subject is missing those inputs (**Table 1**). For additional simulation cases, see **Supplementary Tables S2–S4**.

**Table 1:** Validation for reorient to radiological module.

Cases	Input orientations	Output orientations
Case 1	Lesion: neurological	Radiological
	T1: neurological	Radiological
	Brain: neurological	Radiological
	WM: neurological	Radiological
Case 2	Lesion: radiological	Radiological
	T1: radiological	Radiological
	Brain: radiological	Radiological
	WM: radiological	Radiological
Case 3	Lesion: neurological	Flagged
	T1: neurological	
	Brain: radiological	
	WM: neurological	
Case 4	Lesion: neurological	Flagged
	T1: neurological	
	Brain: radiological	
	WM: radiological	
Case 5	Lesion: neurological	Flagged
	T1: radiological	
	Brain: radiological	
	WM: radiological	
Case 6: Optional	Lesion: neurological	Radiological
input missing	T1: neurological	Radiological
	Brain: missing	Brain extraction set to run on all subjects
	WM: missing	WM segmentation set to run for all subjects

*Simulated cases including subjects with T1, lesion mask (Lesion), brain mask (Brain), and white matter segmentation (WM) inputs with varying orientations. PALS correctly flagged cases in which orientations of input files were mismatched.*

### Lesion Correction for Healthy White Matter Voxels

#### Inter-Rater Reliability

**FIGURE 5:**
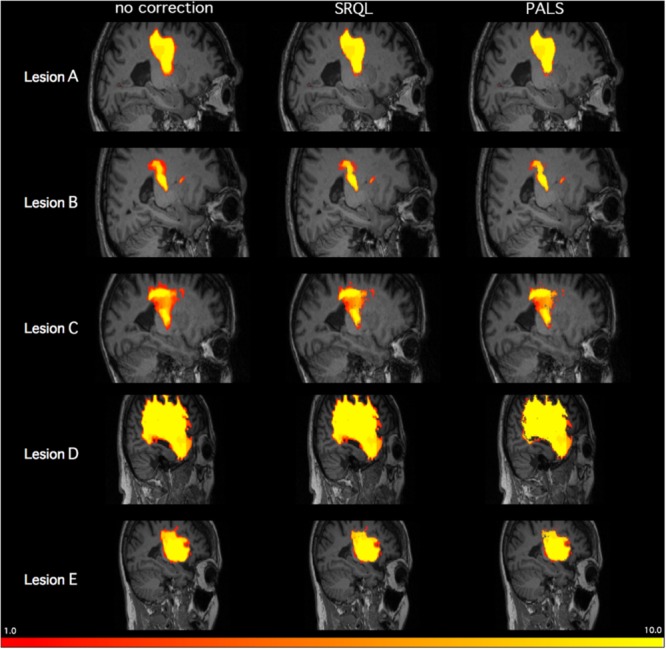
Inter-tracer heat maps of five stroke lesions with and without lesion correction. Brighter colors indicate greater overlap between tracers; as shown above in regions with less bright colors and greater dark red, PALS improves similarity between inter-rater tracings, particularly when lesion correction is included.

For this module, we tested whether PALS could improve inter-rater reliability on five manually segmented lesion masks (**Figure 5**). Ten trained research assistants manually traced stroke lesions from five separate brains with lesions of different sizes (Liew et al., 2018). For each stroke brain, we calculated a dice correlation coefficient (DC) for each pair among manual tracings by 10 different trained individuals to evaluate agreement between all raters. The dice correlation coefficient is a measure of similarity between two images, and is defined as:

$$\begin{array}{c} \mathrm{DC}=2\;*\;\frac{\left|X\cap Y\right|}{\left|X|+|Y\right|} \end{array}$$

where DC ranges between 0 (no overlap) and 1 (complete overlap), and *X* represents voxels in the first lesion volume, and *Y* represents voxels in the second lesion volume. For each stroke brain, the average of all DC values from all 45 pairwise comparisons of manual segmentations was calculated as a mean inter-rater DC score, and then mean inter-rater DC scores across the five stroke brains was again averaged for an overall inter-rater DC score. We then ran both SRQL, our previous version of lesion correction, and PALS-lesion correction on all manual segmentations, using the default value of 5% lesion white matter intensity removal, to compare which performed better, and recalculated the overall inter-rater dice coefficient score on white matter adjusted lesion masks (**Table 2**).

**Table 2:** Inter-rater Dice Correlation Coefficient values with and without lesion correction.

	Lesion A	Lesion B	Lesion C	Lesion D	Lesion E	Average
No correction	0.84 ± 0.006	0.62 ± 0.03	0.66 ± 0.02	0.93 ± 0.002	0.81 ± 0.01	0.77 ± 0.009
SRQL	0.87 ± 0.006	0.63 ± 0.03	0.69 ± 0.01	0.93 ± 0.002	0.82 ± 0.01	0.79 ± 0.009
PALS-LesionCorr	0.87 ± 0.006	0.65 ± 0.03	0.72 ± 0.014	0.94 ± 0.002	0.84 ± 0.009	0.80 ± 0.009

*Average dice coefficient values (mean ± standard deviation) for manual tracings across 10 trained individuals. 1, perfectly overlapping; 0, no overlap. PALS-Lesion correction consistently improved the dice coefficient across all lesions compared to no correction or the earlier SRQL toolbox.*

We next performed a one-way repeated measures ANOVA on the mean inter-rater DC scores averaged over the five lesions to determine whether there were any differences between inter-rater scores without any correction, with lesion correction from SRQL, and with the new PALS lesion correction. Mauchly’s test indicated that the assumption of sphericity had been violated (*p* < 0.001), therefore Greenhouse–Geisser corrected tests are reported (𝜀 = 0.514). We found a significant difference among the inter-rater DC scores (*F* = 5.91, *p* = 0.0183); Tukey *post hoc* comparisons with Bonferroni correction showed that inter-rater DC scores, the average number of voxels overlapping between manual segmentations of the tracers (see above for description), were significantly higher after lesion correction with PALS compared to lesion masks without any adjustment (*z* = 3.43, *p* = 0.0018); other pairwise comparisons did not reach significance (*p* > 0.18). In other words, the lesion correction module in PALS significantly improved the similarity between manual tracings across the 10 tracers. We thus recommend using the PALS lesion correction module when analyzing manually traced lesions.

#### Automated vs. Manual Lesion Segmentations

We were also interested in assessing whether lesion correction could improve similarity between automated segmentations and manual segmentations, the latter considered the gold standard for lesion segmentation. For this evaluation, we used 90 stroke T1-weighted MRIs from the publicly-available ATLAS database (Liew et al., 2018). The ATLAS database consists of chronic stroke (>6 months) MRIs obtained across 11 research groups worldwide, and also includes manually segmented lesion masks for each MRI, created by a team of trained individuals (for further information on the full lesion dataset and labeling protocol, see Liew et al., 2018). The 90 brains included for this evaluation consisted of 34 cortical, 54 subcortical, and 2 cerebellar lesions on both left (*n* = 36) and right (*n* = 54) hemispheres. Lesion volume ranged from 386 to 164,300 mm^3^ (*M* = 31,578.41, *SD* = 38,582.13) based on manual segmentations.

We used the manually segmented lesions included in the ATLAS database as our gold standard of manually traced lesion masks. We then used the lesion identification with neighborhood data analysis (LINDA) approach to automatically segment the 90 stroke T1-weighted MRIs (Pustina et al., 2016). Finally, we calculated the dice DC between each automated segmentation and manually traced lesion and obtained an average DC of 0.58 ± 0.25 (range 0.006 to 0.88).

**FIGURE 6:**
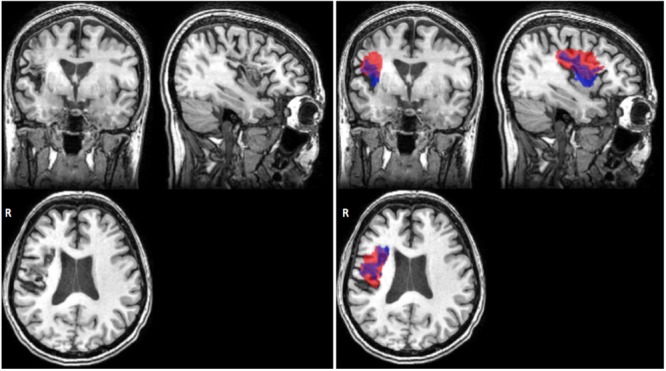
Representative case of automated versus manual lesion segmentation. Left, an individual’s T1w anatomical MRI; Right, the manual lesion mask in blue overlaid on automated lesion mask produced by the LINDA algorithm, in red (Pustina et al., 2016). DC, 0.57 for this lesion.

We note that a DC value of 0.58 is relatively low considering that DC ranges between 0 and 1. However, given that limitations still exist with performance of automated lesion segmentation algorithms, particularly for single-modality data and for data that have been pooled together from different sites, such as the ATLAS database, an average DC of 0.58 is fairly standard (Ito, Kim, and Liew, *under review;* for a representative example, see **Figure 6**).

##### Removing White Matter From Manual Tracings

We next performed lesion correction on manually traced lesions, using the default 5% white matter intensity removal, and re-calculated DC to determine whether lesion correction improved similarity between the manual and automated segmentations. We found that lesion correction on manual lesions made no difference on similarity between manual and automated lesions (average DC before and after correction: 0.58 ± 0.24; *t* = 1.59, *p* = 0.11).

##### Removing White Matter From Automated Segmentations

Finally, we assessed whether lesion correction on automated segmentations could improve similarity to manually traced lesions. We thus applied lesion correction using default values on lesions automatically segmented using LINDA, and calculated DC between manual segmentations (without lesion correction) and white matter corrected automated segmentations for the 90 brain lesions. Here, we found that lesion corrections did not improve similarity between manual and automated lesions and actually significantly decreased similarity by a small amount (average DC before: 0.58 ± 0.03; average DC after correction: 0.57 ± 0.24; *t* = 2.58, *p* = 0.01).

### Lesion Load Calculation and Quality Control

We validated our lesion load calculation module with a CST lesion load calculation tool implemented by a separate research group (Riley et al., 2011). As their group divided up their CST ROI into 16 longitudinal strings to obtain the lesion-to-CST percentage overlap (see Riley et al., 2011 for methods), we also tested the PALS lesion load calculation module with identical ROI input, courtesy of Riley et al. (2011).

For this evaluation, we implemented both lesion load calculation tools on 122 brains from the ATLAS dataset. These brains were made up of 40 cortical, 67 subcortical, 12 brainstem, and 3 cerebellar strokes. To validate that the lesion load tool correctly assesses the hemisphere of the lesion, we included both left (*n* = 70) and right (*n* = 40) hemisphere lesions (and 12 brainstem lesions). Lesion volume ranged from 27 to 62,460 mm^3^ (*M* = 7,391.48 mm^3^, SD = 9,060.82 mm^3^).

We used the left CST ROI, and found a strong significant correlation between the PALS method and the previously described method from Riley et al. (2011) (PALS average CST lesion load percentage: 44.96 ± 44.70%; Riley CST lesion load: 43.75 ± 44.30%; *r* = 0.87, *p* < 0.0001). We also verified that the CST lesion load percentage was equal to 0% on all right hemisphere lesions. However, the correlation was lower than expected. Using our QC tool, we visually inspected the quality of the intermediary outputs created by PALS and identified seven cortical stroke brains that performed poorly on brain extraction and registration. We cleaned up the brain extractions using additional features in FSL’s *BET* (e.g., bias field and neck cleanup), and reran these brains through the PALS pipeline, feeding in the cleaned-up brain extractions (**Figure 4**). As expected, this substantially improved registration. We then re-calculated the CST lesion load as well as the correlation between the values obtained through PALS and the method described above from Riley et al. (2011). Doing so resulted in a stronger correlation between the two lesion load calculation tools (PALS average CST lesion load percentage: 47.33 ± 45.24%; *r =* 0.96, *p* < 0.0001). This demonstrates the importance of performing a thorough quality inspection on each processing step and overall confirms that our tool accurately calculates lesion overlap in accordance with previous work.

We additionally assessed whether the accuracy of lesion load calculation differed between cortical and subcortical lesions. As such, we split these 122 validation cases by category (40 cortical, 67 subcortical, excluding brainstem and cerebellar lesions). We then calculated the Pearson’s correlation coefficient by stroke category, to assess how well the PALS method compares to the method implemented by Riley et al. (2011). For cortical strokes, we obtained a correlation coefficient of *r* = 0.73, *p* < 0.0001; 95% CI [0.54, 0.85]. However, after correcting for image processing errors that occurred for the seven brains mentioned above, we obtained the following values for cortical strokes: *r* = 0.98, *p* < 0.0001; 95% CI [0.97, 0.99]. For subcortical strokes, we obtained a correlation coefficient of *r* = 0.95, *p* < 0.0001; 95% CI [0.93, 0.97]. Again, this demonstrates the susceptibility of larger, cortical strokes to image processing errors and highlights the importance of quality control.

## Discussion

Despite the recent surge of interest in big data neuroimaging, the infrastructure and image processing pipelines necessary to support it, particularly for stroke lesion analysis, are still severely lacking. To this end, we created an open-source toolbox with a user-friendly GUI to help standardize stringent stroke lesion analyses. A detailed manual and source code can be downloaded from our github repository^5^.

To demonstrate some of the key features of the toolbox, we validated use with multi-site data. We demonstrated that PALS successfully harmonizes data to be in the same orientation convention across sites. We also showed that PALS increases inter-rater reliability of manual tracings: applying the lesion correction module in PALS significantly increased similarity between manually segmented lesions compared to no lesion correction and our previous version of lesion correction from the SRQL toolbox. However, we found that similarity between manual segmentations and automated segmentations, in cases where groups might try to use manual segmentations for a subset of the data and automated segmentation in another subset of data, did not improve when applying PALS lesion correction on either the manual segmentations or the automated segmentations. A likely explanation for this is that the automated segmentations algorithm we used (LINDA; Pustina et al., 2016) already included a tissue classification step in the derivation of features, which would prevent white matter voxels to be classified as lesion tissue. We thus recommend that research groups do not mix different lesion segmentation methods (e.g., a subset manually and a subset with an automated algorithm) for the PALS lesion correction module, but rather use lesion correction only for datasets with all manual lesion segmentations. This is because applying white matter intensity removal to human errors in manual segmentations would provide a systematic way to remove voxels within the designated healthy white matter intensity range that might be missed due to human bias (Riley et al., 2011). Finally, we also showed that PALS lesion load calculation module is comparable to another CST lesion load calculator implemented by a different research group.

### Limitations and Future Directions

PALS was created to respond to the need for reliable image processing pipelines for collaborative efforts in stroke neuroimaging. PALS integrates multiple functions into a single analysis pipeline to facilitate lesion analysis and quality control. However, the PALS toolbox has a few limitations. First, as PALS was created to address the need for lesion analysis software that takes a single modality, we have only tested the toolbox on T1w MRI data. We hope to expand these tools for other types of multimodal stroke imaging, such as T2 or FLAIR sequences, in the future. However, we will plan to retain the option for using a single channel input so that users will not be required to have multi-modal data to use PALS. Additionally, in the reorient to radiological module, the PALS toolbox makes the assumption that the input files are in valid NifTI format, which requires proper user input.

We plan to continue to refine our software in the future based on feedback and comments from users^6^, and hope to expand these tools for other multimodal stroke imaging data types. We hope our toolbox will be useful to clinicians and researchers, and foster greater collaboration leading to the discovery of new clinical insights.

## Author Contributions

KI implemented the toolbox, tested the toolbox, and drafted the manuscript. AK implemented the toolbox. AZ-P tested the toolbox and revised the manuscript. SC contributed to the conceptualization of toolbox modules and revised the manuscript. S-LL conceptualized the toolbox, tested the toolbox, and revised the manuscript.

## Conflict of Interest Statement

The authors declare that the research was conducted in the absence of any commercial or financial relationships that could be construed as a potential conflict of interest.

## Footnotes

1 https://github.com/npnl/PALS

2 https://github.com/npnl/PALS

3 https://fsl.fmrib.ox.ac.uk/fsl/fslwiki/Orientation%20Explained

4 https://fsl.fmrib.ox.ac.uk/fsl/fslwiki/FSLeyes

5 https://github.com/npnl/PALS

6 https://github.com/npnl/PALS/issues
