# Supplementary material for: Pipeline for Analyzing Lesions After Stroke (PALS)
Source: Front Neuroinform. 2018 Sep 24;12:63. doi: 10.3389/fninf.2018.00063 (PMC6165891; doi:10.3389/fninf.2018.00063)
Supplement: Supplementary file 1 [file Table_1.DOCX]

Supplementary Material

Pipeline for Analyzing Lesions after Stroke

Kaori L. Ito^1^, Amit Kumar^1^, Artemis Zavnliangos-Petropulu^1^, Steven C. Cramer^2^, & Sook-Lei Liew^1*^

*** Correspondence:** Sook-Lei Liew: sliew@usc.edu

# Table S1. Datasets used to validate reorientation to radiological module

| **cohort** | **subjects** | **Lesion mask / T1 input orientations** | **Final orientation after PALS** |
| --- | --- | --- | --- |
| c0001 | 6 | Orientations mismatched | subjects flagged |
| c0002 | 25 | Orientations mismatched | subjects flagged |
| c0003 | 55 | RADIOLOGICAL | RADIOLOGICAL |
| c0004 | 34 | NEUROLOGICAL | RADIOLOGICAL |
| c0005 | 30 | NEUROLOGICAL | RADIOLOGICAL |
| c0006 | 12 | NEUROLOGICAL | RADIOLOGICAL |
| c0007 | 36 | RADIOLOGICAL | RADIOLOGICAL |
| c0008 | 32 | NEUROLOGICAL | RADIOLOGICAL |
| c0009 | 12 | NEUROLOGICAL | RADIOLOGICAL |
| c0010 | 47 | RADIOLOGICAL | RADIOLOGICAL |
| c0011 | 15 | NEUROLOGICAL | RADIOLOGICAL |
| LTW | 50 | RADIOLOGICAL | RADIOLOGICAL |
| total | 354 |  |  |

We used data from twelve different sites, consisting of a total of 355 MRIs. For all subjects in the first two datasets, the lesion mask and T1 anatomical images were in mismatched orientations; these were correctly flagged by PALS. PALS successfully converted the remaining neurological images to radiological orientation.
