# Supplementary material for: Pipeline for Analyzing Lesions After Stroke (PALS)
Source: Front Neuroinform. 2018 Sep 24;12:63. doi: 10.3389/fninf.2018.00063 (PMC6165891; doi:10.3389/fninf.2018.00063)
Supplement: Supplementary file 3 [file Table_3.DOCX]

Supplementary Material

Pipeline for Analyzing Lesions after Stroke

Kaori L. Ito^1^, Amit Kumar^1^, Artemis Zavnliangos-Petropulu^1^, Steven C. Cramer^2^, & Sook-Lei Liew^1*^

*** Correspondence:** Sook-Lei Liew: sliew@usc.edu

# Table S3. Simulated cases using T1, lesion mask, and brain mask inputs

| Cases | Input Orientations | Output Orientations |
| --- | --- | --- |
| Case 1 | Lesion: neurological | radiological |
|  | T1: neurological | radiological |
|  | Brain: neurological | radiological |
| Case 2 | Lesion: radiological | radiological |
|  | T1: radiological | radiological |
|  | Brain: radiological | radiological |
| Case 3 | Lesion: neurological | flagged |
|  | T1: neurological |  |
|  | Brain: radiological |  |
| Case 4 | Lesion: neurological | flagged |
|  | T1: radiological |  |
|  | Brain: radiological |  |
| Case 5: optional input missing | Lesion: neurological | radiological |
|  | T1: neurological | radiological |
|  | Brain: missing | Brain extraction will run for all subjects |
